# Is self-assessment of medical abortion using a low-sensitivity pregnancy test combined with a checklist and phone text messages feasible in South African primary healthcare settings? A randomized trial

**DOI:** 10.1371/journal.pone.0179600

**Published:** 2017-06-22

**Authors:** Deborah Constant, Jane Harries, Kristen Daskilewicz, Landon Myer, Kristina Gemzell-Danielsson

**Affiliations:** 1 Women’s Health Research Unit, School of Public Health and Family Medicine, University of Cape Town, Cape Town, South Africa; 2 Division of Epidemiology & Biostatistics, School of Public Health and Family Medicine, University of Cape Town, Cape Town, South Africa; 3 Department of Obstetrics and Gynecology, Karolinska Institutet, Stockholm, Sweden; Gynuity Health Projects, UNITED STATES

## Abstract

**Objective:**

To evaluate feasibility of self-assessment of medical abortion outcome using a low-sensitivity urine pregnancy test, checklist and text messages. The study assessed whether accurate self-assessment required a demonstration of the low-sensitivity urine pregnancy test or if verbal instructions suffice.

**Methods:**

This non-inferiority trial enrolled 525 adult women from six public sector abortion clinics. Eligible women were undergoing medical abortion at gestations within 63 days. Consenting women completed a baseline interview, received standard care with mifepristone and home-administration of misoprostol. All were given a low-sensitivity urine pregnancy test and checklist for use 14 days later, sent text reminders, and asked to attend in-clinic follow-up after two weeks. Women were randomly assigned 1:1 to an *instruction-only group* (n = 262; issued with pre-scripted instructions on the low-sensitivity pregnancy test), or a *demonstration group* (n = 263; performed practice tests guided by lay health workers). The primary outcome was accurate self-assessment of incomplete abortion, defined as needing additional misoprostol or vacuum aspiration. Analysis was by intention to treat and a non-inferiority margin was set to six percentage points. Women’s acceptability of their abortion procedure and preferences for follow-up were also assessed.

**Results:**

Follow-up was 81% for abortion outcome, confirmed in-clinic at two weeks or self-reported within six months. Non-inferiority of instruction-only to a demonstration was inconclusive for accurate self-assessment (risk difference for *instruction-only –demonstration*: -2.5%; 95%CI: -9% to 4%). Comparing instruction-only to demonstration groups, 99% and 100% found the pregnancy test easy to do; and 91% and 93% respectively chose the pregnancy test, checklist and text messages for abortion outcome assessment in the future.

**Conclusion:**

Routine self-assessment using a low-sensitivity pregnancy test, checklist and text messages is feasible and preferred by women attending South African primary care abortion clinics. Counselling with additional emphasis on prompt recognition of ongoing pregnancies is recommended.

**Trial registration:**

ClinicalTrials.gov NCT02231619

## Introduction

Early medical abortion with mifepristone and misoprostol was introduced into public sector primary care services in South Africa in 2010 [1]. The proportion of women not returning for in-clinic follow-up after medical abortion has increased in recent years as the regimen is highly effective [2], and the additional visit can present a burden in terms of cost and time [3–7]. Recognizing this, the World Health Organization (WHO) technical guidelines stated that routine in-clinic assessment is not required, if given adequate counselling on signs of ongoing pregnancy and complications [8].

However, published literature indicated that self-assessment of medical abortion based on symptoms alone was not reliable [6, 9, 10], and recently the WHO recommended that self-assessment of medical abortion outcome by women should utilize suitable pregnancy tests and checklists [11]. Multi-level (hCG detection thresholds >25, >100, >500, >2000 and >10,000 milli-international units/mL) and dual-level low-sensitivity pregnancy tests (hCG detection thresholds >5 and >1000 or >2000 milli-international units/mL), supplemented by checklists have shown promising results in the US [12], Europe [4, 13–16] and some low- and middle income countries [3, 6, 17, 18]. The multi-level test, while highly accurate, involves comparing serial hCG measurements performed at baseline and repeated 7–14 days later [17]. As such, both complexity of the test and cost could be a barrier in resource-constrained settings in developing countries. For the South African primary healthcare sector, a single, simple, low-sensitivity urine pregnancy test (LSUPT) for home-use, together with a checklist, might be a more feasible tool for self-assessment of medical abortion outcome. This could be supplemented by automated text message reminders to women over the course of the abortion process, providing an effective, low-cost support mechanism [19]. However, it was unknown if this package would be preferred to in-clinic follow-up by women, and if lack of privacy at home would present a barrier to this approach.

In addition, it was unknown whether women would perform and accurately interpret the LSUPT result without guidance. Although recently developed LSUPTs such as the *check*ToP^®^ (single hCG detection threshold >1000 milli-international units/mL) are similar to the better–known high sensitivity urine pregnancy tests and simpler to use than earlier LSUPTs, previous studies using symptom history [10], multi-level [12] and dual-level pregnancy tests [5] indicated a need for training or detailed instruction on the use of these tools. Accordingly, for this study, investigators included a control group in which participants did a guided practice of the LSUPT and a comparative group who received a brief set of verbal instructions. The comparative arm was thought to be a more feasible service delivery option in the South African public sector.

The aim of this study was to determine the feasibility of self-assessment of medical abortion outcome using a low-sensitivity urine pregnancy test and checklist at 14 days, combined with pre-programmed text messages as a service delivery option for women attending South African primary level abortion clinics. A non-inferiority randomized trial was performed to determine whether instruction-only on the use of a LSUPT for self-assessment of medical abortion outcome would be accurate within six percentage points compared to an assisted practice demonstration.

## Materials and methods

The study was approved by the University of Cape Town Human Research Ethics Committee.

### Study participants

The study (ClinicalTrials.gov, NCT02231619) was conducted at six public sector primary level abortion clinics, including one youth clinic for women under 25 years, in Cape Town, South Africa. To be eligible for the study, women needed to be clinically eligible for medical abortion using mifepristone with home-use of misoprostol, have a gestational age within 63 days, be 18 years of age or older, willing to receive abortion-related text messages on their phone over the next 14 days, able to give informed consent and willing to attend a follow-up visit at the same abortion clinic 2 to 3 weeks later.

### Randomization and allocation

An investigator not involved with the study provided the computer-generated, fieldworker-specific randomization lists with blocks of varying size 8–18. Allocation was in a 1:1 ratio and allocation slips were packed into sequentially numbered opaque sealed envelopes by an administrator uninvolved with the study. Fieldworkers opened the envelope corresponding to the participant enrollment number on conclusion of the baseline interview and conducted the intervention according to the assigned study group. Thus fieldworkers were blinded to the study group allocation only for the baseline interview; for all other study activities, fieldworkers, study investigators and participants were not blinded. Clinic staff were blinded at all times to group assignment of participants.

### Study procedures

**Fig 1:**
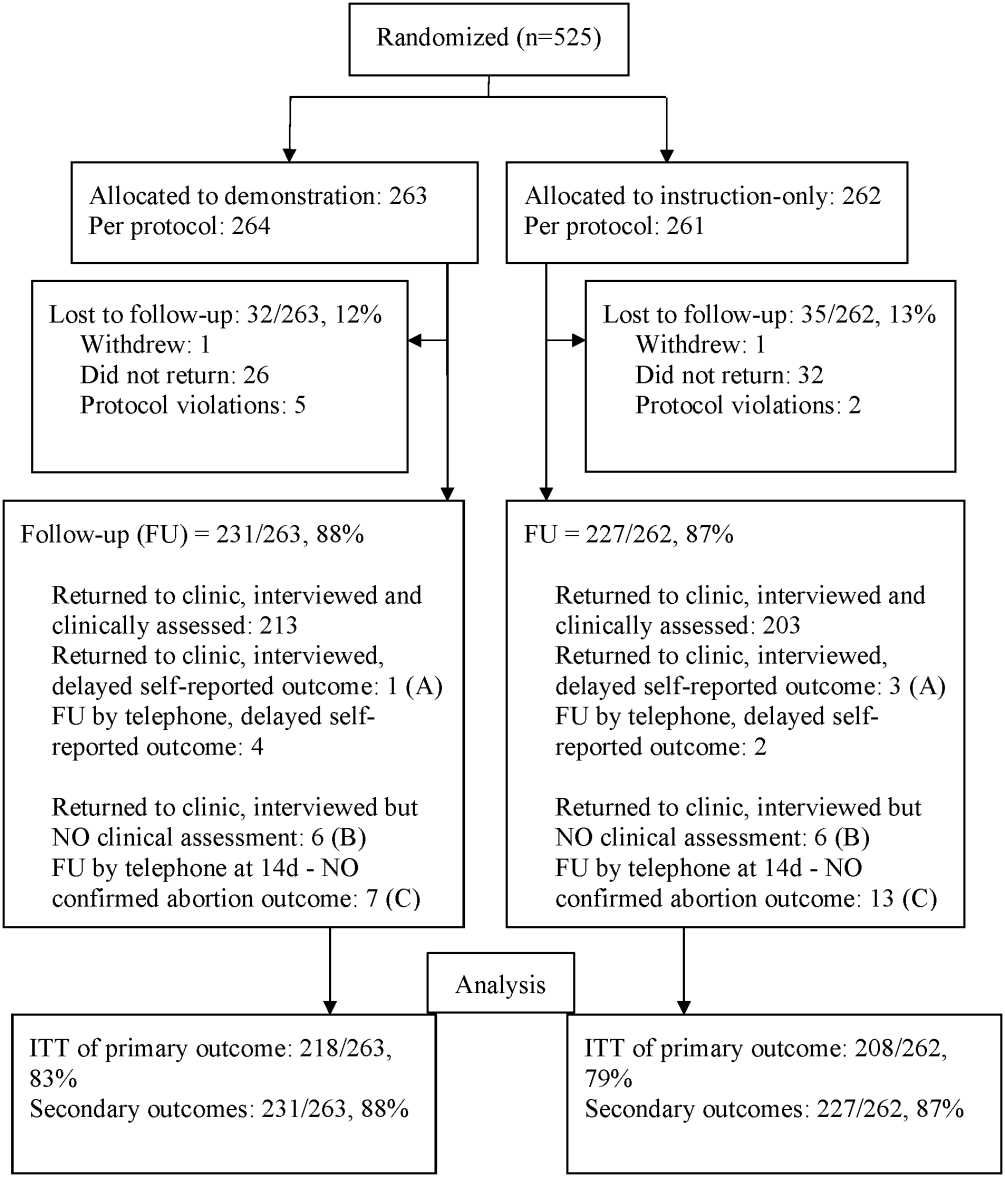
Trial profile. (A)Women returned to the clinic, but the provider was not present. In final FU phone contact abortion outcome was self-reported. (B)Women returned to the clinic, but the provider was not present. Final FU phone contact was unsuccessful and abortion outcome could not be confirmed. (C)Women did not return to the clinic, FU at 14 days was by phone. Final FU phone contact was unsuccessful and abortion outcome could not be confirmed.

From mid-September 2014 to mid-June 2015, study fieldworkers approached clinically eligible women in the waiting area to determine interest in the study. Screening of interested women for study eligibility and administration of written informed consent was then performed in a private area. Eligible, consenting women were enrolled and interviewed using a structured questionnaire, and on completion were allocated to a randomization group (Fig 1). Participants allocated to the demonstration group then conducted the *check*ToP^®^ LSUPT (Veda Lab, France) on their own urine sample and interpreted the result at 5 minutes, guided by a study fieldworker using a standardized procedure and pre-scripted instructions. The instruction-only group were given the same pre-scripted verbal instructions, but were not offered a practice demonstration. All participants were issued with a *check*ToP^®^ test kit to be used on their first morning urine specimen at 14 days after mifepristone and a symptom checklist. Participants then received standard medical abortion care with 200 mg of oral mifepristone, and 800 mcg of misoprostol, to be taken sublingually and buccally, at home 24–48 hours later. Standard care at most clinics was to offer a hormonal contraceptive method, usually depot medroxyprogesterone acetate (DMPA) injectable or the contraceptive implant immediately following mifepristone administration, in accordance with WHO recommendations [8]. Study investigators registered participants’ phone numbers on the day of enrollment into a computer system which sent 19 timed, automated text messages over the next 14 days including reminders on storing the LSUPT away from direct heat, taking their misoprostol, what abortion symptoms to expect, managing pain, responding to excessive bleeding and other complications, conducting the pregnancy test and attending follow-up appointments [19, 20].

Participants were followed-up from October 2014 through July 2015. Fieldworkers interviewed participants attending in-clinic follow-up prior to provider assessment. Interim clinic visits and phone calls, the abortion experience, acceptability of text messages, checklist and LSUPT results (participants drew their LSUPT result into an outline provided on their checklist) and women’s assessment of their pregnancy status were recorded. Women were also asked to interpret their checklist as showing that their “abortion was complete” or whether there was “need for additional abortion care”. Women then saw a nurse provider for clinical assessment of their abortion outcome, including a clinical history and examination. For some women a high sensitivity pregnancy test and/or an ultrasound exam were performed. Providers were instructed by study investigators not to deviate from their standard care in evaluating abortion completion for study participants. On completion of clinical assessment, fieldworkers recorded participants’ preference for follow-up method and post-abortion contraceptive method, provided study participation compensation of ZAR100 (~USD 7) and extracted information on participants’ abortion outcome from clinical records. Participants not attending in-clinic follow-up were contacted the next day by phone. Following three calls and two text messages, if no contact was made, they were considered potentially lost to follow-up (LTF). One final attempt was made to contact all those potentially LTF prior to ceasing all participant tracing end July 2015. Participants completing the follow-up interview by phone received ZAR 50 compensation as phone airtime.

### Study outcomes

The primary study outcome related to feasibility, and was defined as accurate self-assessment of incomplete medical abortion (true positives + true negatives)/total; incomplete medical abortion was defined as the need for surgical intervention or additional misoprostol at in-clinic follow-up. Self-assessment of incomplete abortion was based on women’s interpretation of their checklist to indicate need for additional care and/or a positive LSUPT result. Unclear LSUPT results were combined into a “positive or unclear” category. Accuracy of self-assessment of ongoing pregnancy compared to all other abortion outcomes was also calculated. Secondary feasibility outcomes included performance of the test no less than 10 days after mifepristone and ease of doing the test. Other secondary outcomes were preferences for in-clinic or self-assessment of abortion outcome, satisfaction with the abortion procedure (four-point scale), acceptability of the procedure (would you recommend the procedure to a friend, would you have the same procedure again if you need another abortion—binary scale), and post-abortion contraceptive uptake.

### Sample size

To test our hypothesis that instruction-only was non-inferior to a demonstration of a LSUPT for accurate self-assessment of abortion outcome, we set the margin of non-inferiority to an absolute difference of six percentage points. Given a demonstration of the test, in combination with a checklist, we expected that 95% women would accurately assess their abortion outcome. The non-inferiority margin and this assumption were based on published studies [12, 14, 21], local providers’ opinions, feasibility of implementing the test into service provision, as well as feasibility and cost of conducting the study. We calculated that a total sample size of 416, with 208 in each group, would achieve 80% power to detect a non-inferiority margin difference between the demonstration and instruction-only group of 6% with a one-sided significance level of 0.025. With a potential for loss to follow-up (LTF) of 20%, at least 520 participants were needed for enrollment at baseline.

### Statistical analysis

Statistical analysis was performed using Stata v.13 (Tx). Study groups were compared at baseline using Chi-squared tests. All study outcomes were analysed by intention-to-treat. Abortion outcomes were not evaluated from phone interviews where participants reported no clinic attendance, unless they self-reported abortion completion during a later phone call three to six months after their procedure. Risk ratios were calculated for accurate assessment of incomplete abortion and of ongoing pregnancy and non-inferiority of the instruction-only group was evaluated using the risk difference 95% confidence interval (CI). Sensitivity, specificity, negative and positive predictive values with 95% CIs were also calculated for abortion outcomes. Secondary outcomes were analysed for all participants completing the follow-up interview in person or by phone; groups were compared using Chi-squared tests, with p-values of <0.05 considered statistically significant. Two sensitivity analyses were done by imputing missing values: firstly, with all missing values assigned as correctly self-assessing an incomplete abortion and secondly with all missing values assigned as incorrect self-assessment.

## Results

Recruitment of participants took place between September 2014 and June 2015. Five hundred and twenty-five women were enrolled into the trial, with 263 randomized to the demonstration group and 262 to the instruction-only group (Fig 1). The final number of participants exceeded the estimated minimum sample size by 5 as at (final trial day-1) there were <520 enrolled into the study; at close of (final trial day) N enrolled was 525. There were no significant differences between the groups at baseline indicating the randomization was successful (Table 1). There was 1 withdrawal per group and 7 protocol violations: 2 (demonstration) and 1 (instruction-only) were deemed ineligible for medical abortion following randomization, 3 (demonstration) and 1 (instruction-only) reported having a manual vacuum aspiration (MVA) within 1 week of mifepristone due to minimal or no bleeding following misoprostol, however their pregnancy status was not recorded at the time.

**Table 1:** Participant characteristics at enrollment by randomization group.

Characteristic	Demonstration n = 263	Instruction-only n = 262
Age (years) n(%)		
18–24	101 (38.4)	98 (37.4)
25–29	72 (27.4)	76 (29.0)
>=30	90 (34.2)	88 (33.6)
Home language n (%)		
isiXhosa	214 (81.4)	217 (82.8)
Afrikaans	10 (3.8)	6 (2.3)
English	25 (9.5)	28 (10.7)
Other African language	14 (5.3)	11 (4.2)
Travel time to clinic (minutes) n(%)		
0–15	53 (20.2)	60 (22.9)
16–30	141 (53.6)	135 (51.5)
>=31	69 (26.2)	67 (25.6)
Education level n (%)		
<Grade 12	116 (44.1)	129 (49.2)
>=Grade 12	147 (55.9)	133 (50.2)
Paid work n (%)	120 (46.1)	118 (45.2)
Formal housing n (%)	138 (52.5)	139 (53.1)
Prior pregnancies n (%)		
0	52 (19.8)	42 (16.0)
1	100 (38.0)	96 (36.6)
2+	111 (42.2)	124 (47.3)
Prior abortion n (%)	13 (4.9)	18 (6.9)
Prior medical abortion n (%)	6 (2.3)	8 (3.1)
Gestational age (days) n (%)		
28–48	122 (46.4)	102 (38.9)
49–63	141 (53.6)	160 (61.1)
Did own high-sensitivity pregnancy test prior to clinic visit (HSUPT) n/N (%)	217/259 (83.8)	222/257 (86.4)

Clinic follow-up was scheduled at 14–21 days; among non-returnees with whom phone contact was initially unsuccessful, self-reported abortion outcomes were documented three to six months later by investigators in a final attempt at contact. The primary outcome was confirmed for 218 in the demonstration and 208 in the instruction-only group. Some participants completed their follow-up interviews at 14–21 days, but did not have their abortion outcome clinically assessed at the time of the interview. They were included in the analysis of secondary outcomes. This larger sample consisted of 231 in the demonstration and 227 in the instruction-only group (Fig 1).

For the primary outcome, 25/263(17%) in the demonstration group and 54/262 (21%) in the instruction-only group were LTF. There were no significant differences in baseline characteristics between those LTF and those retained for the demonstration group. In the instruction-only group, younger women (median age = 25 vs. 28 years; p = 0.277, Kruskal-Wallis test), and those living in better housing (68.5% vs. 49.0%; p = 0.011) were more likely to be LTF.

There were no differences between study groups in the numbers of additional visits or calls made to the clinics nor in reasons for those visits and calls. Overall, 3% (15/458) of women contacted the clinic prior to their scheduled appointment, and none received additional abortion care at the unscheduled visit. The most common reason for the additional contact was scant bleeding (9/15, 60%). Other reasons included side effects or the need for reassurance that the abortion was proceeding normally. There was one adverse event in the instruction-only group in which the participant sought hospital admission for symptoms of dizziness and weakness some hours after taking her misoprostol. She was stabilized in hospital, did not receive a blood transfusion, or any abortion-related treatment. She completed her self-assessment and follow-up clinic visit as scheduled.

Table 2 shows provider assessment of abortion outcome and self-assessment results. There was no significant difference between study groups for provider-assessed ongoing pregnancy (demonstration group: 2/218, 0.9%; instruction-only group: 1/208 0.5%. p = 1.000, two-sided Fisher’s exact test). Numbers of incomplete abortions requiring additional provider care for either ongoing pregnancy, retained products or persistent bleeding were also not significantly different between demonstration and instruction-only groups (19/218, 8.7%; 18/208, 8.7%. p = 0.982).

**Table 2:** Provider assessment and self-assessment of abortion outcome.

	Ongoing pregnancy		Incomplete abortion (including. ongoing pregnancy)		Complete abortion		p-value*
	Demonstration	Instruction-only	Demonstration	Instruction-only	Demonstration	Instruction-only	
Provider assessment	2/218 (0.9%)	1/208 (0.5%)	19/218 (8.7%)	18/208 (8.7%)	199/218 (91.3%)	190/208 (91.4%)	0.982^#^
Self-assessment: PT** + checklist	N/A	N/A	30/231 (13.0%)	37/227 (16.3%)	201/231 (87.0%)	190/227 (83.7%)	0.316^#^
Self-assessment: checklist only	N/A	N/A	24/231 (10.4%)	23/227 (10.1%)	207/231 (89.6%)	204/227 (89.9%)	0.928^#^
Self-assessment: PT** only	Ongoing pregnancy		No ongoing pregnancy				
	21/231 (9.1%)	28/208 (12.3%)	210/231 (91.9%)	180/208 (86.5%)			0.261

^#^ Incomplete vs. complete abortion

*P-values for Chi-squared tests comparing study groups.

**PT = pregnancy test

There were no significant differences between study groups for abortion outcomes by self-assessment using the LSUPT and checklist, the checklist only or the LSUPT only (Table 2). Of the 21/231 (9.1%) and 28/227 (12.3%) participants in the demonstration and the instruction group who reported a positive or unclear LSUPT result, 15 and 18 reported a faint second line positive for the LSUPT, and 6/231 (2.6%) and 10/227 (4.4%) reported clear double line positive results (p = 0.292).

**Fig 2:**
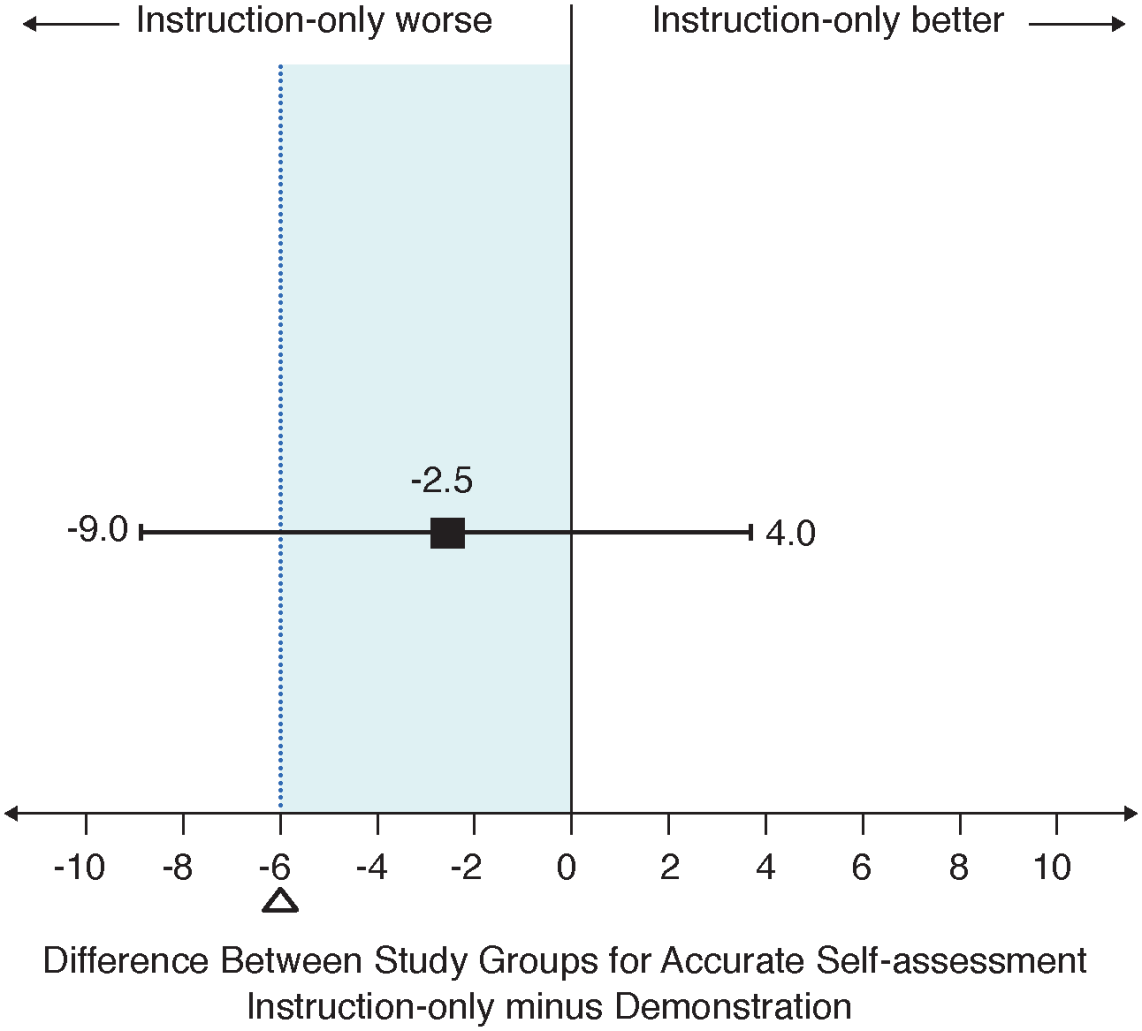
Observed difference (95% CI) between study groups for accurate self-assessment.

The study primary outcome, accurate self-assessment of incomplete abortion, was reported for 191/218 (87.6%, 95% CI: 83.2–92.0) in the demonstration group and for 177/208 (85.1%, 95% CI: 80.3–89.9) in the instruction-only group. The risk ratio (RR) was not statistically significant (RR = 1.03; p = 0.449), however the risk difference of -2.5% (95%CI: -9.0 to 4.0) was beyond the non-inferiority margin by three percentage points and overlapped zero (Table 3), indicating an inconclusive result for non-inferiority (Fig 2). The two sensitivity analyses did not alter this result (data not shown). Testing for heterogeneity using the Mantel-Haenszel method showed no effect for age (p = 0.670), prior use of a high sensitivity pregnancy test (p = 0.175), home language (p = 0.090), housing type (p = 0.441), education (p = 0.968), gestational age (p = 0.185) or prior abortion (p = 0.232). Accurate self-assessment of ongoing pregnancy by LSUPT, was also similar for the two groups (RR = 1.04; p = 0.277). As with the primary outcome, the 95% confidence limit for the risk difference was outside the non-inferiority margin and crossed zero (-3.3%; 95% CI: -9.4 to 2.7), signifying that non-inferiority for instruction-only was inconclusive (Table 3).

**Table 3:** Comparison between study groups for 1) Self-assessment of incomplete abortion with low-sensitivity pregnancy test and checklist and 2) Self-assessment of ongoing pregnancy with low-sensitivity pregnancy test.

Test statistic	1) Incomplete abortion: Provider: vs. self-assessment: low-sensitivity pregnancy test + checklist			2) Ongoing pregnancy: Provider vs. self-assessment: low-sensitivity pregnancy test only		
	Demonstration	Instruction-only	Risk	Demonstration	Instruction-only	Risk
Accuracy n/N	191/218	177/208	-2.5%	197/218	181/208	-2.9%
% (95%CI)	87.6% (83.2–92.0)	85.1% (80.3–89.9)	(-9.0 to 4.0)	90.4% (87.4–95.0)	87.0% (83.9–92.7)	(-8.7 to 2.9)
Sensitivity n/N	11/19	12/18		1/2	1/1	
% (95% CI)	57.9% (33.5–79.7)	66.6% (41.0–86.8)		50% (1.3–98.7)	100% (2.5–100)	
Specificity n/N	180/199	165/190		196/216	180/207	
% (95% CI)	90.5% (85.5–94.2)	86.8% (81.2–91.3)		90.7% (86.1–94.3)	87.0% (81.6–91.2)	
Predictive Value (+)	11/30	12/37		1/21	1/28	
% (95% CI)	36.7% (19.9–56.1)	32.4% (18.0–49.8)		4.8% (0.1–23.8)	3.6% (0.1–18.3)	
Predictive Value (-)	180/188	165/171		196/197	180/180	
% (95% CI)	95.7% (91.8–98.1)	96.5% (92.5–98.7)		99.5% (97.2–100.0)	100% (98.0–100.0)	

Sensitivity and specificity, negative and positive predictive values had overlapping 95% CIs for the two study groups for self-assessment of incomplete abortion and for ongoing pregnancy (Table 3). Of the 3 ongoing pregnancies, there was 1 false negative test and 1 true positive test for ongoing pregnancy in the demonstration group and 1 true positive test in the instruction-only group. Taking both study groups together, self-assessment of incomplete abortion using the combined LSUPT and checklist did not significantly impact on accuracy compared to using the LSUPT alone (Chi^2^ = 1.077, p = 0.299, data not shown).

There were no significant differences between study groups for secondary feasibility outcomes, follow-up preferences, satisfaction and acceptability of abortion procedure or post-abortion contraceptive uptake (Table 4). Almost all (99.6%) in both study groups did the LSUPT. Most (99.0%) did the LSUPT at 10 days or later after their mifepristone. Ninety-nine percent found the test easy or very easy to do and 98.5% preferred self-assessment to an in-clinic follow-up appointment. Most were satisfied with their abortion (97.8%), would recommend the method to a friend (82.0%) and would want the same method, should they need another abortion (86.2%). Ninety-six percent in both groups started a contraceptive method, of whom just over half in both groups initiated the method at their mifepristone clinic visit, (known as “quick-start” contraception). Most commonly the quick-start methods were injectable short-term methods, followed by the contraceptive implant, with some participants receiving oral contraceptive pills (data not shown).

**Table 4:** Feasibility and acceptability of self-assessment and abortion experience.

Outcome	Demonstration	Instruction-only	p-value*
Did pregnancy test at home n (%)	230/231 (99.6)	226/227 (99.6)	0.990
Did pregnancy test at >=10 days n/N (%)	206/208 (99.0)	199/201 (99.0)	0.973
Easy or very easy to do pregnancy test n/N (%)	230/231 (99.6)	224/227 (98.7)	0.307
Preferred follow-up method for future use			
Return for in-clinic assessment n/N (%)	3/231 (1.3)	4/227 (1.8)	0.686
Pregnancy test, with or without checklist & SMS, contact clinic if need to n/N (%)	228/231 (98.7)	223/227 (98.2)	
Satisfied or very satisfied with abortion n/N (%)	225/231 (97.4)	222/226 (98.2)	0.545
Would recommend abortion method to a friend n/N (%)	193/229 (84.3)	180/226 (79.7)	0.199
Would have same procedure, if needed abortion again n/N (%)	197/230 (85.7)	195/225 (86.7)	0.754
Started post-abortion contraception	221/231 (95.7)	217/227 (95.6)	0.580
Contraception started at mifepristone visit n/N (%)	118/221 (53.4)	115/217 (53.0)	0.580

* P-values for Chi-squared tests

## Discussion

This study suggests that self-assessment of early medical abortion using a LSUPT supplemented by automated text messages, with or without a checklist, could be a feasible option for women accessing abortion care at primary care level in South Africa. However non-inferiority of self-assessment of medical abortion given instruction-only compared to a demonstration of the test cannot be inferred from our findings. Taking into account the high percentage of provider intervention for indications other than ongoing pregnancy (34/426; 8.0%), and the preponderance of faint line positive tests observed among those assessed by provider to have complete abortions (29/389; 7.5%), self-assessment accuracy was generally good. Combining both the study groups together, 88.7% (95% CI: 85 1–91.7) self-assessed that their procedure was complete with no need for additional treatment (specificity), representing the proportion of women who could safely forgo in-clinic follow-up. In addition, 88.9% (95% CI: 85.5–91.7) of study participants accurately self-assessed no-ongoing pregnancy (specificity). This is lower than reported for the multi-level urine pregnancy test (MLUPT) [6, 18], but similar to other studies using the LSUPT [4, 13].

The false negative LSUPT result in the demonstration study group is concerning. A single false negative result was reported in a larger study using the MLUPT [6], and studies from other settings using a LSUPT have consistently reported occasional false negative test results for ongoing pregnancies [3–5]. In these studies, as in ours, all women with a false negative test for ongoing pregnancy received their abortion despite the delay incurred. Given the occasional false negative test result, when combined with the LSUPT, a better-designed checklist or information sheet on signs of ongoing pregnancy might serve as a risk management tool. If used, the number of unnecessary clinic follow-up visits could be expected to increase, however this would constitute a small percentage. Critics of the checklist have suggested that providers might simply instruct women to return if they experience pregnancy symptoms or have a positive test [18]; an approach that may be preferred, depending on contextual factors.

It was unexpected that prior experience using a high sensitivity test did not improve self-assessment of ongoing pregnancy in either study group. If the LSUPT were implemented in South Africa, specific counselling is likely to be needed on performing and interpreting the test and the checklist to ensure women are adequately prepared to self-assess their abortion outcome. Current standard abortion care in South African public sector clinics involves pre-abortion group counselling by providers. This might serve as an opportune moment for a simulated demonstration on the use and interpretation of the LSUPT. In addition, for women preferring self-assessment, but unsure of doing this, the group session offers the chance for them to benefit from other’s support. Women’s confidence in self-assessing their medical abortion may increase over time as this approach becomes better known generally. Providers’ trust and support for women’s choice of self-assessment is also likely to grow as this becomes more commonplace.

The text message program used in this study was previously shown to be effective in preparing women for what to expect and well-suited to supplementing self-managed abortion care by providing a continuum of timed information and reminders on signs of complications or a failed abortion procedure (19). This intervention has the advantage of providing information and triggers to action within timeframes matched to the individual users and the content and number of messages can easily be modified if preferred. While this form of intervention depends on adequate telecommunications infrastructure, phone ownership and familiarity with text messages, these are characteristic of the South African setting generally, and among South African women seeking abortion, specifically [20, 22].

Despite the promising results for the LSUPT in this study, health system factors need to be taken into account for implementation of the LSUPT to be a feasible service delivery option. Currently, neither the *check*ToP^®^ nor the other more affordable LSUPTs are commercially available in South Africa. The estimated cost of the *check*ToP^®^ used in this study combined with the low rate of ongoing pregnancy may weigh against introducing this into routine care in resource-limited settings providing free abortion care. Ensuring that women are able to complete their abortion through timely and effective referral to facilities providing safe surgical abortion may be a more appropriate approach to strengthening abortion services overall.

Our checklist was designed to assist women to self-assess symptoms of incomplete abortion such as scant or persistent bleeding. However a limitation of the study was that the primary outcome was accurate self-assessment of incomplete abortion but the groups differed only with respect to orientation to the pregnancy test. In designing the study, the investigators took the pragmatic decision to use incomplete abortion for the study primary outcome, to allow for an adequately powered study. Given that the incomplete abortion rate was the same between study groups, as were the self-assessment outcomes from the checklist, we believe that the study results can be accepted as valid.

There was differential LTF between study groups, with participants missing not-at-random which could have biased our findings. However, the sensitivity analyses performed suggest robustness in the study results. In addition, in determining sample size, we estimated that 95% of women in the demonstration arm would accurately self-assess their abortion outcome. In the study, this was found to be 88%, and thus the study was slightly underpowered. A larger sample size might have resulted in a more conclusive result comparing the study groups. Our study population is representative of the urban population attending free public sector health facilities in South Africa, classified as a high-middle income country. Fifty-three percent of participants had completed high school education, all had use of a mobile phone, and all were able to read text messages in their home-language, thus our study results are likely to be generalizable to similar settings elsewhere in South Africa and globally.

## Conclusion

Providing brief verbal instructions compared to a guided demonstration on the use of a low-sensitivity urine pregnancy test did not impact conclusively on accurate self-assessment of abortion outcome. From women’s perspectives, the LSUPT is feasible and acceptable for use in South Africa. The inclusion of automated text reminders is well-liked by women and when combined with the low-sensitivity urine pregnancy test, is preferred to clinic follow-up post-abortion.

## Supporting information

S1 File Data file. (XLSX)

S2 File Consort checklist. (DOCX)

S3 File Study protocol. (DOCX)
