# Supplementary material for: Is self-assessment of medical abortion using a low-sensitivity pregnancy test combined with a checklist and phone text messages feasible in South African primary healthcare settings? A randomized trial
Source: PLoS One. 2017 Jun 22;12(6):e0179600. doi: 10.1371/journal.pone.0179600 (PMC5480887; doi:10.1371/journal.pone.0179600)
Supplement: S2 File — (DOCX) [file pone.0179600.s002.docx]

CONSORT Statement 2006 - Checklist for Non-inferiority and Equivalence Trials

**Items to include when reporting a non-inferiority or equivalence randomized trial**

| PAPER SECTION And topic | Item | Descriptor | Reported on  Page # |
| --- | --- | --- | --- |
| TITLE & ABSTRACT | 1 | [How participants were allocated to interventions](http://www.consort-statement.org/index.aspx?o=1107) (e.g., "random allocation", "randomized", or "randomly assigned"),  specifying that the trial is a non-inferiority or equivalence trial. | 1,2 |
| INTRODUCTION Background | 2 | [Scientific background and explanation of rationale](http://www.consort-statement.org/index.aspx?o=1016),  including the rationale for using a non-inferiority or equivalence design. | 4,5 |
| METHODS Participants | 3 | [Eligibility criteria for participants](http://www.consort-statement.org/index.aspx?o=1017%22%20%5Cl%20%223a) (detailing whether participants in the non-inferiority or equivalence trial are similar to those in any trial(s) that established efficacy of the reference treatment) and the [settings and locations where the data were collected](http://www.consort-statement.org/index.aspx?o=1017%22%20%5Cl%20%223b). | 5,6 |
| Interventions | 4 | [Precise details of the interventions intended for each group detailing whether the reference treatment in the non-inferiority or equivalence trial is identical (or very similar) to that in any trial(s) that established efficacy, and how and when they were actually administered](http://www.consort-statement.org/index.aspx?o=1021). | 6,7 |
| Objectives | 5 | [Specific objectives and hypotheses](http://www.consort-statement.org/index.aspx?o=1022), including the hypothesis concerning non-inferiority or equivalence. | 5 |
| Outcomes | 6 | [Clearly defined primary and secondary outcome measures](http://www.consort-statement.org/index.aspx?o=1023%22%20%5Cl%20%226a) detailing whether the outcomes in the non-inferiority or equivalence trial are identical (or very similar) to those in any trial(s) that established efficacy of the reference treatment and, when applicable, any [methods used to enhance the quality of measurements](http://www.consort-statement.org/index.aspx?o=1023%22%20%5Cl%20%226b) (e.g., multiple observations, training of assessors). | 8 |
| Sample size | 7 | [How sample size was determined](http://www.consort-statement.org/index.aspx?o=1024%22%20%5Cl%20%227a) detailing whether it was calculated using a non-inferiority or equivalence criterion and specifying the margin of equivalence with the rationale for its choice. When applicable, [explanation of any interim analyses and stopping rules](http://www.consort-statement.org/index.aspx?o=1024%22%20%5Cl%20%227b) (and whether related to a non-inferiority or equivalence hypothesis). | 8,9 |
| Randomization -- Sequence generation | 8 | [Method used to generate the random allocation sequence, including details of any restrictions](http://www.consort-statement.org/index.aspx?o=1025) (e.g., blocking, stratification) | 6 |
| Randomization -- Allocation concealment | 9 | [Method used to implement the random allocation sequence](http://www.consort-statement.org/index.aspx?o=1026) (e.g., numbered containers or central telephone), clarifying whether the sequence was concealed until interventions were assigned. | 6 |
| Randomization -- Implementation | 10 | [Who generated the allocation sequence, who enrolled participants, and who assigned participants to their groups](http://www.consort-statement.org/index.aspx?o=1027). | 6 |
| Blinding (masking) | 11 | [Whether or not participants, those administering the interventions, and those assessing the outcomes were blinded to group assignment](http://www.consort-statement.org/index.aspx?o=1028%22%20%5Cl%20%2211a). If done, [how the success of blinding was evaluated](http://www.consort-statement.org/index.aspx?o=1028%22%20%5Cl%20%2211b). | 6 |
| Statistical methods | 12 | [Statistical methods used to compare groups for primary outcome(s)](http://www.consort-statement.org/index.aspx?o=1029%22%20%5Cl%20%2212a), specifying whether a one or two-sided confidence interval approach was used. [Methods for additional analyses](http://www.consort-statement.org/index.aspx?o=1029%22%20%5Cl%20%2212b), such as subgroup analyses and adjusted analyses. | 9 |
| RESULTS  Participant flow | 13 | [Flow of participants through each stage](http://www.consort-statement.org/index.aspx?o=1018) (a diagram is strongly recommended). Specifically, for each group report the numbers of participants randomly assigned, receiving intended treatment, completing the study protocol, and analyzed for the primary outcome. [Describe protocol deviations from study as planned, together with reasons](http://www.consort-statement.org/index.aspx?o=1086). | 10  Figure 1 |
| Recruitment | 14 | [Dates defining the periods of recruitment and follow-up](http://www.consort-statement.org/index.aspx?o=1087). | 10,12 |
| Baseline data | 15 | [Baseline demographic and clinical characteristics of each group](http://www.consort-statement.org/index.aspx?o=1088). | Table 1 |
| Numbers analyzed | 16 | [Number of participants (denominator) in each group included in each analysis and whether the analysis was](http://www.consort-statement.org/index.aspx?o=1089) “intention-to-treat” and/or alternative analyses were conducted. State the results in absolute numbers when feasible (e.g., 10/20, not 50%). | 12  Figure 1 |
| Outcomes and estimation | 17 | [For each primary and secondary outcome, a summary of results for each group, and the estimated effect size and its precision](http://www.consort-statement.org/index.aspx?o=1090) (e.g., 95% confidence interval). For the outcome(s) for which non-inferiority or equivalence is hypothesized, a figure showing confidence intervals and margins of equivalence may be useful. | 12-14  Tables 2,3,4 |
| Ancillary analyses | 18 | [Address multiplicity by reporting any other analyses performed](http://www.consort-statement.org/index.aspx?o=1091), including subgroup analyses and adjusted analyses, indicating those pre-specified and those exploratory. | 15 |
| Adverse events | 19 | [All important adverse events or side effects in each intervention group](http://www.consort-statement.org/index.aspx?o=1092). | 14 |
| DISCUSSION Interpretation | 20 | [Interpretation of the results](http://www.consort-statement.org/index.aspx?o=1019), taking into account the non-inferiority or equivalence hypothesis and any other study hypotheses, sources of potential bias or imprecision and the dangers associated with multiplicity of analyses and outcomes. | 19-21 |
| Generalizability | 21 | [Generalizability (external validity) of the trial findings](http://www.consort-statement.org/index.aspx?o=1094). | 21 |
| Overall evidence | 22 | [General interpretation of the results in the context of current evidence](http://www.consort-statement.org/index.aspx?o=1095). | 19,20 |

**www.consort-statement.org**
