# Supplementary material for: Is self-assessment of medical abortion using a low-sensitivity pregnancy test combined with a checklist and phone text messages feasible in South African primary healthcare settings? A randomized trial
Source: PLoS One. 2017 Jun 22;12(6):e0179600. doi: 10.1371/journal.pone.0179600 (PMC5480887; doi:10.1371/journal.pone.0179600)
Supplement: S3 File — (DOCX) [file pone.0179600.s003.docx]

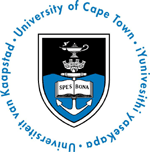
 **
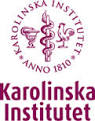
**

**THE USE OF A LOW SENSITIVITY PREGNANCY TEST COMBINED WITH PHONE TEXT MESSAGES TO ASSESS EARLY MEDICAL ABORTION OUTCOME IN THE SOUTH AFRICAN PUBLIC SECTOR SETTING**

**PROTOCOL VERSION (3.0)**

**30 June2014**

**Project to be sponsored by**

**Safe Action Abortion Fund**

**Collaborating Institutions**

Women’s Health Research Unit, School of Public Health & Family Medicine, University of Cape Town, South Africa

Department of Obstetrics & Gynecology, Karolinska Institutet, Sweden

**Principal Investigator**

Deborah Constant. MPH, MSc(Anat),BSc(Phys)

Senior Researcher

Women’s Health Research Unit

School of Public Health & Family Medicine

University of Cape Town Faculty of Health Sciences

Anzio Road

Observatory 7925

SOUTH AFRICA

P +2721 4066722

M +2772 252 7415

F +2721 4066788

Deborah.constant@uct.ac.za

**Co-Investigators**

Kristina Gemzell-Danielsson

| Marijke Alblas |  |  |
| --- | --- | --- |

**TABLE OF CONTENTS**

[I. ABBREVIATIONS 4](#_Toc409695648)

[II. PROTOCOL SYNOPSIS 5](#_Toc409695649)

[III. BACKGROUND AND RATIONALE 10](#_Toc409695650)

[IV. AIM AND OBJECTIVES 12](#_Toc409695651)

[V. STUDY POPULATION 12](#_Toc409695652)

[a. Number of subjects 12](#_Toc409695653)

[b. Inclusion criteria 12](#_Toc409695654)

[c. Study sites 13](#_Toc409695655)

[d. Recruitment 13](#_Toc409695656)

[VI. STUDY INTERVENTIONS 13](#_Toc409695657)

[a. Refresher training 13](#_Toc409695658)

[b. Study intervention. 14](#_Toc409695659)

[VII. STUDY PROCEDURES 14](#_Toc409695660)

[a. Overview 14](#_Toc409695661)

[b. Enrollment 14](#_Toc409695662)

[c. Minimizing loss to follow-up 16](#_Toc409695663)

[VIII. SAMPLE SIZE 17](#_Toc409695664)

[IX. ANALYSIS 17](#_Toc409695665)

[X. CRITERIA FOR DISCONTINUATION 17](#_Toc409695666)

[XI. DATA MANAGEMENT 18](#_Toc409695667)

[XII. DURATION OF PROJECT 18](#_Toc409695668)

[XIII. ETHICS 18](#_Toc409695669)

[a. Informed consent and confidentiality 18](#_Toc409695670)

[b. Risk assessment 19](#_Toc409695671)

[c. Additional ethical concerns 19](#_Toc409695672)

[d. Protocol violations 20](#_Toc409695673)

[e. Participant compensation. 20](#_Toc409695674)

[XIV. RECORD RETENTION 20](#_Toc409695675)

[XV. RESULTS DISSEMINATION ANDPROJECT SUSTAINABILITY 20](#_Toc409695676)

[XVI. REFERENCES: 21](#_Toc409695677)

# ABBREVIATIONS

HCG Human chorionic gonadotrophin

LSUPT Low sensitivity urine pregnancy test

MA Medical abortion

MVA Manual vacuum aspiration

PI Principle investigator

SMS Short Message System

UCT HREC University of Cape Town Human Research Ethics Committee

WHO World Health Organization

WHRU Women’s Health research Unit

# PROTOCOL SYNOPSIS

| Title | The use of a low sensitivity pregnancy test combined with phone text messages to assess early medical abortion outcome in the South African public sector setting. |
| --- | --- |
| Background and Motivation | Early medical abortion was introduced in the South African public sector recently and is expected to address some of the existing barriers to abortion care, in particular the lack of trained of providers of surgical methods. The follow-up in-facility visit to assess outcome and provide family planning is recommended by the World Health Organization to be discretionary and there is considerable loss-to follow up. The study will explore the feasibility and safety of an alternative to in-facility follow-up. |
| Study setting | The project will be conducted in the public health sector at two study sites in the Cape Town metro district. We will identify sites that provide services to disadvantaged communities, targeting youth clinics in these areas.  If proved feasible, this follow-up alternative can reduce both time and cost burdens for providers as well as for women having medical abortion who prefer not to come to the clinic for follow-up. This is particularly relevant for remote and underserved regions in the country. It is also expected that this approach will find greatest acceptability among younger women. If successful, the results can inform future provincial and national guidelines on provision of early medical abortion. |
| Design | Feasibility study with nested randomization component |
| Aim and Objectives | AIM: To strengthen medical abortion service provision and offer a safe alternative to the in-facility follow-up visit using a low sensitivity pregnancy test combined with phone text messages for women undergoing early medical abortion  OBJECTIVES   1. To clarify and standardize best practice relating to clinical management at follow-up by providers in accordance with international guidelines. 2. To design a self-assessment package that could be the preferred option to a follow-up visit at the clinic by the majority of women having early medical abortion. 3. To improve the accuracy of self-assessment of need for additional care following medical abortion, by combining a low sensitivity urine pregnancy test with self-assessment using text on mobile phones. 4. To compare accuracy of self-assessment given only verbal instructions versus conducting a directly observed test at baseline. 5. To improve knowledge of the advantages of long acting reversible methods as well as other modern contraceptive options among women undergoing early medical abortion and who prefer to self-assess their abortion outcome. |
| Study Interventions | A refresher training session for providers by an expert advisor on clinical assessment of medical abortion outcome, use of ultrasound and indications for additional treatment and treatment recommendations  Programmed text messages of support and information for participants on what to expect, alerts to complication and family planning information, a symptom checklist and a low sensitivity urine pregnancy test. |
| Sample size | A minimum of 416 participants completing follow-up interviews.. We expect loss to follow up of up to 20% |
| Main Inclusion Criteria | Each subject must meet the following essential criteria:   - First trimester pregnancy up to 63 days gestation age - Undergoing abortion with mifepristone and misoprostol - Over 18 years of age - Willing to receive abortion-related messages on her phone - Ability to give informed consent - Willingness to attend follow-up visit at clinic |
| Informed consent and confidentiality | The informed consent process will be administered in a private location at the clinic by the study fieldworker in a face to face interview.  Each participant will be asked to sign her name on the consent form, and will be assigned an ID number. The name and ID number will be listed together in the Study Register along with her phone number. Only her Study ID and phone number and the date will be registered onto the text message delivery system. Thereafter instruments used for data collection will list the Study ID number only.  Confidentiality and data security will be maintained throughout the project through careful control of hard copy documents and electronic data. Access to these materials will be limited to the study team, and the Study Coordinator and Principal Investigator will accept ultimate responsibility |
| Procedures | Women will be approached individually in the clinic waiting room in a discrete and private way by the study field worker and will be offered participation. Enrolment will take place following mifepristone ingestion. Enrolment involves informed consent, eligibility assessment, allocation to study group,conducting baseline low sensitivity pregnancy test or receiving verbal instruction on doing the test, an interview and registration of participant’s phone number onto the study system.  Over the next 2 weeks the participant will receive text messages. On the morning of her follow-up visit (usually at 14 days) she will conduct the low sensitivity pregnancy test at home, and record the result. At the facility follow-up visit she will be interviewed before assessment by the clinic clinician. Data will be collected on her interpretation of the pregnancy test, and acceptability of the messages. Following clinical assessment, this information will be recorded on the study forms and participant’s preferred follow up option will be recorded. |
| Compensation for participants | Participants will receive R100 compensation for their time at their follow-up clinic visit or R50 if they do the follow-up interview by phone |
| Potential harms and protection of privacy | Potential medical risks to clients participating in the study are the same as those they would incur by undergoing a medical abortion outside of the study. No additional medical risks are associated with participation in the proposed research. There is a small potential risk that participants who successfully complete the self-assessment may feel that there is no need to return for follow up. 2 reminder SMSs will be sent to encourage in-person return to the clinic. Also 3 attempts will be made to trace the participant by phone and encourage her to return. We also offer participant compensation for time and costs at the follow-up interview.  At enrolment, potential study participants will be asked to consider privacy. The consent form says:  “How private is your phone?  The SMSs that we send you will mention things like termination, taking pills, and contraception. So if others use your phone and you don’t want them to know about your abortion, you must either make sure that you don’t share your phone for 2 weeks from today, or you should delete any SMSs that you don’t want anyone else to read.”  This, coupled with low rates of shared phones in South Africa, should be sufficient to deal with privacy concerns. However if the woman agrees to receive SMSs and then later realizes that her privacy can be compromised, she is able to send a free SMS to prevent her receiving any further study SMSs. |
| Statistical Analyses | The outcome of preferred follow-up method will be evaluated by percentages with 95% confidence intervals, stratified by facility. Standard descriptive measures (means and standard deviation, proportions and interquartile ranges) will be used for univariates. Validity of the pregnancy test and checklist against the clinical assessment of need for follow-up care will be done using sensitivity, specificity and predictive values. Accuracy of self-assessment of the need for follow-up care will be compared for the 2 study groups using Chi squared analysis.  Acceptability of the text messages and family planning uptake will be assessed using descriptive measures. Associations of outcomes with age, education, language, relative proximity to clinic and abortion experience will be explored using multiple regression methods. |

# BACKGROUND AND RATIONALE

Despite the enactment of South Africa’s liberal abortion law in 1996, implementation of these policies remains uneven, and in the last decade evidence suggests that the initial gains in service provision made in the first decade are being eroded (1). This can largely be attributed the scarcity of trained providers offering manual vacuum aspiration (MVA) (Harries (2). Medical abortion (MA) has the potential to increase women’s access to abortion, as it has a higher degree of acceptability than surgical methods to both providers and clients in South Africa and early MA can be provided in a broader base of primary care services. MA in the first trimester of pregnancy using mifepristone and misoprostol was approved in 2001 and provided in the private sector in South Africa since 2002. The service was extended into the public sector in 2010 starting in the Western Cape and expanding gradually to the rest of the country. Wide-spread availability of medical abortion can address some of the existing barriers related to lack of trained of providers of MVA.

When first developed, it was recognized that early medical abortion using mifepristone and a prostaglandin analogue would have particular relevance in low resource countries where there is a lack of providers with surgical expertise (3). The provision of medical abortion requires counseling on the timing and administration of the drugs, correct management of on-going pregnancy and possible complications such as unusually heavy bleeding as well as supportive guidance on what to expect during the home phase of the procedure and future contraception choices. Backup vacuum aspiration is required only for a small percentage of women. Thus it is well-suited to lower levels of the health care system and lower- and middle-income (LMIC) settings where there is an appropriate referral system (4) but where there may be limited providers with surgical expertise.

Advantages of medical over surgical methods include privacy, control of the process by the client, avoidance of surgical instrumentation of the uterus and the potential for increased access to services (5,6). Potential disadvantages are a higher number of visits, limited eligibility in terms of gestational age, pain, cramping and bleeding, and unsafe loss to follow-up (7) and lost opportunities for contraception uptake where there is loss-to-follow-up.

Specific challenges that women face with MA include the in-person follow-up visit requirement which imposes a burden on their time and cost resources, coping with the abortion experience emotionally and physically in cases where social support is absent and developing self-efficacy in preventing unwanted future pregnancies, particularly if they forego the follow up visit (8)

The World Health Organization’s (WHO) most recent guidelines state that the follow-up visit is not required but may be discretionary (9), however it is still the standard of care in both the private and the public sector in South Africa, although increasingly women do not attend this visit (8,10). The default rate on follow-up in the Western Cape, South Africa varies from 15% to more than 30% (manuscript under submission, M-Assist study, HREC REF 477/2010). At the follow-up visit the outcome of the medical abortion is assessed by clinical examination in most cases including an ultrasound scan. While ultrasound is useful to detect cases of ongoing pregnancy, where there are only retained products or a gestational sac visible, it often leads to unnecessary interventions. Over-treatment with either MVA or additional misoprostol and further follow-up visits is common in South Africa (unpublished data, M-Assist study, HREC REF 477/2010).

As mobile phone penetration in South Africa is extremely high and most women own a mobile phone, calling women who default on their follow-up visit on their mobile phones has been moderately successful in local NGOs and has been implemented in other country settings (11). However this solution is resource-intensive on the provider side (personal communication: MA provider May, 2010), and is not feasible in the resource-constrained South African public sector setting. Also, concerns have been expressed about health worker abuse of phones (personal comm. Sub-district Manager for Health, Western Cape, 2010).

In a recent study in the Western Cape, South Africa (8) a randomized controlled trial was conducted comparing standard of care to an intervention in which clients undergoing medical abortion received information and support messages on their mobile phones; used a self-assessment questionnaire via mobile and received family planning reminders and accessed family planning information via mobile. Publications on this study have been submitted for publication. Results showed that the support messages were effective in reducing anxiety relating to the abortion process and that there was an increase in uptake of long acting reversible contraception among women receiving the text messages compared to the control group. Most women were able to complete the self-assessment on their mobile phones, however the assessment did not correctly identify failed procedures in 2 out of the 3 cases where an additional surgical procedure was performed at follow-up. Inadequate identification of ongoing pregnancy using self-assessment questionnaires or checklists alone is a consistent finding in recent research (10,12). Other studies (13,14) have shown that a combination of self-assessment questions with either a low sensitivity urine pregnancy test (LSUPT) which indicates when urine human chorionic gonadotrophin (HCG) levels are 1,000 mIU/ml or greater, or a semi-quantitative pregnancy test (SQPT) is the most promising strategy to date for providing an alternative to a follow up appointment. A self-administered pregnancy test should be as simple as possible to use, as robust as possible and require as little training as possible. The LSUPT fits these criteria better than the SQPT used by Blum et al (13) which is too complicated to be handled by many women. As no tests have been shown to be 100% accurate, a self-assessment checklist should also be provided as additional means to identify whether additional facility care may be required.

This protocol describes a feasibility study which will describe the use of a self-assessment package for women undergoing MA that combines a low sensitivity urine pregnancy test and checklist with text messages that provide support, information and family planning tips. The study will determine whether this is a preferred method of assessment to in-facility follow-up and whether women are able to accurately assess their need or not of additional in-facility care.

# AIM AND OBJECTIVES

AIM: To strengthen medical abortion service provision and offer a safe alternative to the in-facility follow-up visit using a low sensitivity pregnancy test combined with phone text messages for women undergoing early medical abortion

OBJECTIVES

1. To clarify and standardize best practice relating to clinical management at follow-up by providers in accordance with international guidelines.
2. To design a self-assessment package that could be the preferred option to a follow-up visit at the clinic by the majority of women having early medical abortion.
3. To improve the accuracy of self-assessment of need for additional care due to ongoing pregnancy or an incomplete procedure, by combining a low sensitivity urine pregnancy test and checklist with text messages on mobile phones.
4. To compare accuracy of self-assessment given only verbal instructions versus conducting a directly observed test at baseline.
5. To increase access of MA clients to information about contraceptive choices.

# STUDY POPULATION

The project will be conducted in the public health sector at 4 or 5 study sites in the Cape Town metro district. We will identify sites that provide services to disadvantaged communities, targeting at least 1 youth clinic in these areas.

## Number of subjects

The project will recruit at least 500 participants . We expect loss to follow up of approximately 20 %

## Inclusion criteria

Screening for eligibility for the abortion procedure will be done by clinic staff following the standard of care at the study site and is not part of this protocol. To be included in the study, each potential participant should meet the following essential criteria:

- She is in the first trimester of pregnancy up to a maximum of 63 days gestation age.
- She is undergoing abortion with mifepristone and will take misoprostol at home.
- She is over 18 years of age.
- She is willing to receive abortion-related messages on her phone over the next 14 days.
- She demonstrated the ability to give informed consent.
- She is willing to attend follow-up visit at the same abortion clinic.

## Study sites

We will select study sites where MA using mifepristone and misoprostol is an established standard of care and where follow-up is encouraged and is estimated to be less than 25%, and where post abortion contraception is provided. Suitable sites should be carrying out approximately 30 procedures per month or more. We will target at least 1 youth clinic. Final selection of sites to be included will be contingent on approval being granted by the provincial or city management authorities.

## Recruitment

Participants will be recruited from women who are eligible for MA according to the clinic, have received the pre-abortion counseling that is routinely offered by the clinic and have chosen to proceed with their MA. The routine pre-abortion counseling should include instruction on the requirement to attend the follow-up visit at the facility.

# STUDY INTERVENTIONS

## Refresher training

A refresher training session for providers by an expert advisor will be held at the participating study sites. The content will cover clinical assessment of medical abortion outcome, use of ultrasound and indications for additional treatment and treatment recommendations. This will be offered to all clinic staff before participant recruitment commences. New providers employed at the study sites during the duration of the study will also be offered this training. Where appropriate, providers from other provincial or city clinics may attend, given permission by their managers. The scheduling for this session will be arranged in negotiation with the study site facility manager and should be convenient and not interfere with any standard clinic activities and patient care.

## Study intervention.

The intervention will consist of the following

1. A baseline and a follow-up LSUPT to be carried out by the participant.
2. . Participants will be randomized to 2 groups. Women in Group A will conduct a baseline LSUPT on their own urine specimen with help from the fieldworker. Group B will receive a standardized set of verbal instructions on how to do the test. Both groups will do a LSUPT at home on Day 12.
3. A paper-based instruction sheet for the LSUPT (as a reminder) and symptom checklist
4. Timed text messages delivered automatically by short message systems (SMSs) to participant’s cell phone over 14 days commencing the day after the participant takes the mifepristone. The messages will guide them through the MA process with information around medication effects and their expected timing, what to expect, and will use a supportive tone. No additional burden will be placed on health providers. The SMSs will be scheduled onto a web-based system by the study interviewer as soon as the woman has been recruited to the study. Specifically, the SMSs will include these topic areas:
   - Reminders to take misoprostol, and how to time that.
   - What to expect after taking it, and when (e.g. bleeding, cramping, nausea).
   - Reminder to note down when bleeding starts and how much (how many pads used).
   - Recognising problems (too much bleeding, and fever).
   - How long bleeding can go on for.
   - Reminders to do LSUPT.
   - Contraception short tips.
   - Reminders to access contraception information from a mobisite (m.ichoosewhen) or MXIT.

# STUDY PROCEDURES

## Overview

Each participant will be enrolled into the study at the clinic once she has decided to proceed with MA and either before or immediately after taking the mifepristone. A second interview will be conducted at her follow-up clinic visit approximately 14 days later. Study procedures are described below

## Enrollment

***Eligbilty.*** The study interviewer will introduce the study individually to all women undergoing MA and establish interest and eligibility to participate.

***Informed consent.*** The interviewer will administer the informed consent to interested and eligible women in a private location. She will explain the purpose, potential benefits and risks especially regarding phone privacy. If the woman wishes to participate, she must sign the consent form which has been approved by the UCT HREC. No data may be collected until after this form is signed.

***Contact information***. The interviewer will enter the participant’s phone number, her study number and her choice of language for SMSs onto the computer message delivery system

m.ichoosewhen mobisite

Participants will be shown how to access the mobisite on their phones and will asked to bookmark it if they have smart phones. They will be also asked to browse the mobisite m.ichoosewhen on an electronic tablet while waiting for their procedure. This is to ensure that participant can exercise informed choice if providers administer contraceptive methods at the same clinic visit that women take their mifepristone***.***

***Allocation to study Group:*** The interviewer will allocate the participant to study Group A or Group B by opening the sequentially numbered opaque sealed envelope containing the allocation

***: Group A.*** The interviewer will assist the participant in conducting a baseline LSUPT (ToP Check, VEDALAB, France) on a fresh urine sample and help her interpret the result. The interviewer will show the participant picture mock ups of possible LSUPT that she might observe when she does the test at home..

***Group B*** The interview will give the participant verbal instructions on how to conduct the pregnancy test from a prepared script.All participants will be provided with a sample cup, a LSUPT, a checklist and written instructions on doing the test.

***Baseline interview.*** The interviewer will conduct a short (approximately 15 minutes) baseline interview and extract information from her medical chart which includes information on the following:

- Socio-demographic information
- Reproductive history
- Contraceptive history and future intentions
- Gestation age
- Feelings and expectations around this abortion
- Phone privacy and usage

***Follow-up interview.*** This interview will take place at the clinic, prior to the participant being assessed by the clinician. If the participant did not do the LSUPT, she will be asked to do a test at this time point, in private, in the clinic bathroom. The interview will take approximately 20 minutes and will record information on the following:

- Result of LSUPT
- Interpretation of result of LSUPT
- Difficulty or not doing and interpreting LSUPT
- Current health status (bleeding fever, lower abdominal pain)
- Results of the symptom checklist
- Abortion experience
- Feedback on SMSs
- Contraception intentions

When the participant has completed her clinic assessment, the interviewer will ask the participant which method of follow-up was preferred, will reimburse the participant for her time and record data from her medical chart on the clinical assessment and any additional treatment recommended by the clinician. Women who do not return for their follow-up visit will be contacted by phone to complete the follow-up interview telephonically. These women will receive compensation in the form of airtime.

In addition, in-depth interviews will be conducted with the first 20 women who report that the self-assessment package was hard to use, or who did not manage to correctly self-assess. Following rapid analysis of these interviews, adjustments will be made the self-assessment package if necessary and recruitment will halt until this is done. Thereafter another 10 similar interviews will be conducted and further adjustment done, if necessary. In addition, we will conduct short interviews with providers for a different sample of cases (n~20) where additional clinical management was rendered.

## Minimizing loss to follow-up

A high rate of follow-up is needed for interpretation of the study results. Study staff will therefore attempt up to 3 successful phone contacts (where she is able to speak to the participant) to encourage return to the clinic. If the participant does not return, part of the follow-up interview will be done by phone. Participants who complete this phone interview will be reimbursed R50 in airtime.

# SAMPLE SIZE

The estimated required sample size is based on percentage of women we expect to require additional clinical care at follow-up due to ongoing pregnancy, incomplete abortion, or a complication such as fever, infection or ongoing bleeding. A sample size of 288 will be sufficient to detect the need for additional care in 5% (±2) of women, with a 2sided alpha level of 0.05. With loss to follow-up of 25%, the study should recruit a minimum of 360 women.

For the randomization component of the study: Assuming 95% in Group A accurately self- assess the need for additional cares, a sample size of 208 in each group two will achieve 80% power to detect a non-inferiority margin difference between the group proportions of 6%. In total, 416 participants completing a follow-up assessment are needed. If loss to follow-up reaches 20%, 500 participants will need to be enrolled at baseline.

# ANALYSIS

Preferred follow-up method will be calculated using percentages with 95% confidence intervals, stratified by facility. Standard descriptive measures (means and standard deviation, proportions and interquartile ranges) will be calculated for all relevant variables. Missing values will not be imputed but baseline measures for those lost to follow-up will be calculated to determine any bias that could result from missing data. Acceptability of the SMSs and family planning uptake will be also be assessed using standard descriptive statistical measures.

Accuracy of the pregnancy test with checklist and SMSs against the clinical assessment of need for follow-up care will be determined by calculating sensitivity, specificity and predictive values.

Ease of performing the test and accuracy of the study interventions to self-assess need for follow-up care will be compared for Group A and Group B using Chi squared analysis. For all outcomes, associations with other factors such as age, education, language, relative proximity to clinic and abortion experience will be explored using multiple regression methods.

# CRITERIA FOR DISCONTINUATION

Study participants will be provided, via SMS, with a free number to which they can send an SMS to discontinue receiving further study SMSs. The study participant would then be called on their mobile to find out why they chose to discontinue the SMSs. There should be no reason to terminate the study, as the study does not in any way endanger participants: they still follow the standard of care. However, study participants may chose to leave the study at any point without penalty.

# DATA MANAGEMENT

All interview data will be recorded on paper data forms and all forms will be signed and dated by the interviewer. The study coordinator will be responsible for all data management processes. Questionnaires will be double-entered into an Epidata database, which will be exported to STATA v13 for analysis. Checks for errors in data entry and logical checks for out of range responses, discrepancies in data and other errors will be done in STATA. Completed questionnaires will be stored in a locked cabinet at WHRU under the oversight of the study coordinator and PI.

# DURATION OF PROJECT

| Activity | Months | | | | | |
| --- | --- | --- | --- | --- | --- | --- |
|  | 1-2 | 3 - 6 | 7-9 | 10 - 12 | 13-15 | 16 - 18 |
| Preparatory work  Ethical approval  Finalisation of study instruments | X |  |  |  |  |  |
| Recruit women for study |  | X | X | X |  |  |
| Data analysis |  |  |  | X | X |  |
| Dissemination of results with study sites |  |  |  |  |  | X |

# ETHICS

## Informed consent and confidentiality

Study personnel will obtain written informed consent in the participant’s preferred language prior to enrollment into the study. WHO guidelines were followed in drawing up the consent forms, which were adapted to local content.

All participants will be given information about the study and their rights as part of the informed consent process. If they agree to participate, they will be asked to sign an informed consent form. As part of this informed consent process, potential participants will be informed of the purpose and methods of the study and persons to contact if they have any questions about the study. Researchers will ensure that study participants understand that they are able to refuse to participate in the study and confidentiality of information will be ensured. There will be no direct benefits to the individual women from participation, however taking part in the study may help to make medical abortion services more convenient and accessible for women seeking abortion in future.

Project staff, including interviewers, will be instructed on issues of confidentiality. All data will be managed and reviewed only by researchers conducting the study. Access to information linking individuals and cell phone numbers to data will be restricted to field workers and principal and co-investigators. Presentation of data will exclude all possible identifying links to individuals or phone numbers.

Privacy will be maintained throughout the research process. Participants will be assured of their right to decline to answer questions or withdraw from the study without prejudice.

## Risk assessment

Potential medical risks to clients participating in the study are the same as those they would incur by undergoing a medical abortion outside of the study. No additional medical risks are associated with participation in the proposed research. All research instruments will be piloted to ensure participant comprehension, sensitivity and appropriateness.

Fieldworkers will go extensive training on interviewing techniques as well as orientation to abortion issues in general and those specific to medical abortion. If possible we will employ fieldworkers with some working experience in abortion.

## Additional ethical concerns

Client privacy needs to be respected in abortion service provision, and the study will be designed to minimize the potential of that privacy being compromised. Privacy is of concern in relation to the SMSs as these are received on the client’s phone, and stored until deleted. At enrolment, potential study participants will be asked to consider privacy. The consent form says:

“**How private is your phone?**

The SMSs that we send you will mention things like termination, taking pills, and contraception. So if you share your phone or others use your phone and you don’t want them to know about your abortion, you must either make sure that you don’t share your phone for 6 weeks from today, or you should delete any SMSs that you don’t want anyone else to read.”

This, coupled with low rates of shared phones in South Africa, should be sufficient to deal with privacy concerns. However if the woman agrees to receive SMSs and then later realises that her privacy can be compromised, she is able to send a free SMS to prevent her receiving any further SMSs. From our previous study, we anticipate this will be a very rare occurrence. SMSs will be sent out from a web-based system in which there is no third-party company involved in SMS scheduling and sending. We do not foresee the potential of women responding to the messages as they are sent from a mobile number that is clearly ‘non-human’ and is recognised as being a ‘broadcast message’ in South Africa. We will ensure the necessary deletions of SMSs and phone numbers at the end of the project.

## Protocol violations

The PI will notify the UCT HREC of any protocol violations that involve the informed consent process or subject safety.

## Participant compensation.

Participants will receive R100 compensation for their time and trouble resulting out of participation in the project. They will receive this as cash following completion of their follow-up interview. If they do not return to the clinic and complete the follow-up interview on the phone, they will receive R50 airtime.

# RECORD RETENTION

All record related to the study will be kept for 5 years after the end of the study, whereafter they will be destroyed.

These records include:

- All approvals (ethics and access)
- Informed consent forms signed by subjects
- Completed data collection forms
- Electronic records of participant phone numbers

# RESULTS DISSEMINATION ANDPROJECT SUSTAINABILITY

Within the duration of the study time-line, peer reviewed journal articles will be submitted for publication. Funding will be sought to present the findings at an appropriate international conference.

If these study results demonstrate that this package for self-assessment is acceptable, preferred by the majority of participants, reliable and feasible for South African women, it can provide essential evidence required for such an option to be introduced into practice.

SMSs to provide supplemental information in real-time on the abortion process and on conducting self-assessment have been shown in an earlier randomized controlled trial to be effective, very well received and appreciated by women undergoing early medical abortion in South Africa.(Pan African Clinical Trials Registry (PACTR201302000427144; publications submitted). The improved accuracy for identifying need for further care by including the LSUPT optimizes the package as a safe alternative that also emphases the importance and the options for post abortion contraception. To this end the mobisite will continue to be available as an on-going initiative if funding can be secured.

The policy and practice implications of the study will be of value to all stakeholders in abortion service provision. We will disseminate the results of this work to staff at study sites as well as other facilities in the Western Cape Province that are offering early medical abortion. We also plan to present the results to the Western Cape Provincial Department of Health, the Local Authority management team, the National Department of Health and other representatives interested in strengthening abortion services in order to discuss the findings and identify important next steps.

Specifically by simplifying procedures and at the same time as increasing safety of medical abortion, access to safe abortion can be increased in South Africa. This could be achieved by partnering with government key players and non-governmental organizations operating in the country that provide training and care.

# REFERENCES:

(1) Department of Health. Saving Mothers. Second Report on Confidential Enquiries into
Maternal Deaths in South Africa 2005- 2007. Pretoria. 2009.

(2) Harries J, Stinson K, Orner P. Health care providers' attitudes towards termination of pregnancy: a qualitative study in South Africa. BMC Public Health 2009 Aug 18;9:296.

(3) Rodger MW, Logan AF, Baird DT. Induction of early abortion with mifepristone (RU486) and two different doses of prostaglandin pessary (gemeprost). Contraception 1989 May;39(5):497-502.

(4) Consensus statement. Medical Abortion: Expanding Access to Safe Abortion and Saving Women's Lives. Reprod Health Matters 2005 11;13(26):11-12.

(5) Blanchard K, Blanchard K, Schaffer K, McLeod S, Winikoff B. Medication abortion in the private sector in South Africa. European J. of Contraception and Reproductive Healthcare 2006;11(4):285-290.

(6) Cooper D, Dickson K, Blanchard K, Cullingworth L, Mavimbela N, von Mollendorf C, et al. Medical abortion: the possibilities for introduction in the public sector in South Africa. Reprod Health Matters 2005 Nov;13(26):35-43.

(7) Hassoun D. Medical abortion technologies of 21st century. Presentation at ICMA/IPAS African meeting on Medical Abortion, Johannesburg, South Africa 2009.

(8) Constant D, de Tolly K, Harries J, Myer L. Mobile phone messages to provide support to women during the home phase of medical abortion in South Africa: a randomized controlled trial. (Submitted, Oct 2013)

(9) World Health Organization. Safe Abortion: Technical and Policy Guidance for Health Systems. 2nd ed. Geneva, Switzerland :World Health 0rganization;2012.

(10) Clark W, Bracken H, Tanenhaus J, Schweikert S, Lichtenberg ES, Winikoff B. Alternatives to a routine follow-up visit for early medical abortion. Obstetrics & Gynecology 2010;115(2, Part 1):264.

(11) Perriera LK, Reeves MF, Chen BA, Hohmann HL, Hayes J, Creinin MD. Feasibility of telephone follow-up after medical abortion. Contraception 2010;81(2):143-149.

(12) Jackson AV, Dayananda I, Fortin JM, Fitzmaurice G, Goldberg AB. Can women accurately assess the outcome of medical abortion based on symptoms alone? Contraception 2012 Feb;85(2):192-197.

(13) Blum J, Shochet T, Lynd K, Lichtenberg ES, Fischer D, Arnesen M, et al. Can at-home semi-quantitative pregnancy tests serve as a replacement for clinical follow-up of medical abortion? A US study. Contraception 2012 Dec;86(6):757-762.

(14) Cameron ST, Glasier A, Dewart H, Johnstone A, Burnside A. Telephone follow-up and self-performed urine pregnancy testing after early medical abortion: a service evaluation. Contraception 2012 Jul;86(1):67-73.
